# VCSEL pair used as optical pointers in a contact lens for gaze tracking and visual target designation

**DOI:** 10.1371/journal.pone.0267393

**Published:** 2022-07-11

**Authors:** François-Maël Robert, Vincent Nourrit, Laure Adam, Jean-Louis de Bougrenet de la Tocnaye

**Affiliations:** 1 Optics Department, IMT Atlantique, Brest, France; 2 LCS, Hérouville-Saint-Clair, France; Rutgers University Newark, UNITED STATES

## Abstract

We present a new eye-tracking and target designation device based on a contact lens incorporating a pair of vertical-cavity surface-emitting lasers (VCSELs). We describe the operating principle, the manufacturing process and characterize the impact of the VCSELs encapsulation on their optical properties. We then describe how such device can be incorporated into an eye-wear or a visual augmented system. We compare two different detection set-ups, the first using a camera and the second a position sensitive device, both illustrating different laser beam detection modes. We analyze their performances in terms of angular accuracy, speed, compactness, manufacturability, compared to current conventional eye-tracking systems. We emphasize how the use of two VCSELs and the control of their orientation during the encapsulation can simplify their integration in host systems and improve the gaze detection performance. Finally, we describe various embodiments and discuss potential improvements that can be expected in future systems.

## 1. Introduction

Since the construction of the first eye trackers at the beginning of the 20^th^ century, eye tracking has found applications in a wide variety of fields ranging from psychology to industrial engineering, passing by marketing or neuroscience [1, 2]. Today, eye tracking technology is destined to play a major role in future user interfaces. It can provide feedback on users’ attention and cognitive load [3, 4], or allow optimizing virtual reality (VR) or augmented reality (AR) displays performances by focusing resources where users are looking at [5]. It is particularly suited for future integrated vision augmented systems (e.g. for defense such as Microsoft’s Integrated Visual Augmented Systems (IVAS) [6] or medical applications [7]) allowing the user to control computer functionalities in a more natural way (e.g. replacing mouse by tracked line of gaze [8]) and enabling target designation by visual pointing [9].

In this context, we demonstrated the principle of a new generation of eye-tracking devices that addresses some of the current limitations of existing head mounted ones in terms of accuracy, speed and compactness. Although a wide range of eye tracking techniques exist (see [10] for a short review), mobile eye trackers are mainly video based ones with an accuracy between 0.5–1° and a data rate no higher than 500Hz. In addition, the necessity to have a clear view of the eyes and powerful computers to calculate the gaze direction from the recorded images make them difficult to use in demanding environments (e.g. in surgical theatre when the surgeon wears magnifiers, in constrained environments such as head mounted displays, etc.).

The principle of this new eye-tracking system is based on a contact lens in which an infrared laser is embedded [11]. We denoted this device as the Cyclops lens in reference to the famous X-Men comics hero. The Cyclops lens is a scleral lens made in PMMA, with a total diameter of 16.5 mm. The absence of contact with the cornea makes these lenses comfortable to wear, and they are very steady when worn (unlike usual contact lenses), which is a prerequisite regarding the application. The lens works together with an associated eyewear integrating a primary antenna and a spot detection system. The lens embeds a flexible secondary antenna, an electronic circuit with a VCSEL (at 850 nm) and a near field integrated circuit (NFC) for energy harvesting management and communication. The primary antenna activates the VCSEL, by electromagnetic induction. The detection system on the eyewear detects the VCSEL spot thus allowing to follow eye motions.

A preliminary eye-tracking system has already been demonstrated on a bench prototype, with the Cyclops mounted on a motorized mock-up eye, but with a single VCSEL and not fully encapsulated [11]. From these first experiments we concluded that the use of a single VCSEL was not the best configuration for eye tracking. In contrast, the use of two VCSELs, possibly pointing in different directions could provide several advantages: it would facilitate to determine the center and normal to the pupil, redundancy could improve tracking performances, and it would allow tracking the eye over a wider angular rotation domain. In addition, and most importantly, it would provide more flexibility in the positioning of the spot detectors as close as possible to the eye and out of the field of view.

In this paper, we thus present a new improved version of our Cyclops lenses that we hereinafter refer to as the contact lens pointer (CLP). The electronics embedded in the CLP has been modified, with the removal of the NFC chip and the inclusion of a second VCSEL. Since the whole device relies on detecting the laser spots, the impact of encapsulation on the laser beams is critical. Moreover, two new configurations for the spots’ detection are proposed. In the first one, a camera focuses on the contact lens and the VCSELs act as a marker. In the second, a Position Sensitive Detector (PSD) intercepts the laser beam and the VCSELs are used as pointers. In addition to eye tracking, target designation can be done easily, by a simple command such as a blink or eye scrolling (e.g., switching on/off the visual pointer). The paper is organized as follows: the CLP lens design integrating two VCSELs is presented in section 2. The impact of encapsulation on the laser beams characteristics is studied in section 3. Two configurations of spot detection systems are proposed, and their respective performances in terms of tracking accuracy are measured in section 4. Section 5 presents the manufacturing process to adjust the VCSEL orientation. Two embodiments are suggested in section 6, followed by a discussion on each system and future works in section 7.

## 2. Contact lens double laser pointer description

**Fig 1:**
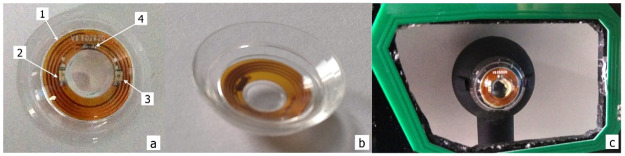
CLP presentation. a) VCSELs pair prototype (1: encapsulated circuit. 2: left VCSEL. 3: right VCSEL. 4: capacitor). b) Back side. c) CLP mounted on the mock-up eye behind the primary antenna (green element) in the eyewear.

The first difference with the Cyclops lens, besides the integration of a second VCSEL [11] is that the electronics is simpler. The NFC chip has been removed, which allowed to encapsulate the secondary antenna in the scleral contact lens, leading to a fully safe and wearable device (Fig 1). This chip is not needed since in the considered application no data transfer is required, and because the two VCSELs can be driven alternatively in a flip-flop mode. The second important difference is thus the presence of a second VCSEL. In terms of power consumption, it is almost the same as with only one VCSEL because both VCSELs are alternatively powered at high frequency (here 13.56 MHz). When the alternative current delivered by the CLP-antenna is negative, one VCSEL lases, while the other one acts as a blocking diode. When the current is positive, the behavior is reversed. Each VCSEL rectifies the HF like a simple diode and lazes for half a cycle so the transformer is loaded in a balanced way and delivers the same current for each cycle. As a result, each VCSEL lases at 13.56 MHz.

We made a series of double laser pointer contact lenses on this principle (Fig 1) that we characterized and used in two detection configurations to assess the detection performances.

## 3. Impact of encapsulation on the VCSEL beam

As previously stated, since the whole device relies on detecting the laser spots it is important to assess how encapsulation may affect the laser beams. First, because the electronics substrate including the VCSELs is initially flat, and its manual encapsulation on a curved surface may result in variations of the pointing characteristics (i.e., the angular separation between each laser). Second, the contact lens part on top of the VCSEL can reduce the transmission, change the beam direction, and increase divergence, which is relevant in the PSD case (since the resolution depends on the spot size). Therefore, it is required to quantify these impacts before designing the complete eye-tracking system and using the CLP. We thus compared these parameters (power, direction, divergence) before and after encapsulation. These measures were carried out on three encapsulated CLP samples and compared with the ones for a non-encapsulated circuit reference.

### 3.1. Impact on transmitted power

The optical power emitted by each VCSEL has been measured by a Thorlabs power meter PM100D before and after encapsulation. The distance between the contact lens and the eyewear was 13 mm, which is a usual eye-glasses distance. The mean emitted power for the six VCSELs before encapsulation was 120±23 μW and 108±28 μW afterwards. The loss is explained partially by the transmittance factor of the PMMA, which is around 93% at 850 nm and dioptric reflections on CLP inner surfaces. This loss is acceptable and does not require increasing the power emitted by the primary antenna, which is of 340 mW.

All lasers worked above lasing threshold (voltage larger than 2.0 V, current superior to 4.0 mA for a minimum lasing power around 20 μW) and the efficiency of the transmission of electrical power by induction was measured at 2.4%. (The poor coupling efficiency is mainly due to the limited number of windings and the geometric mismatch between two antennas, cf. Fig 1c. This could be improved by modifying the primary antenna or using a transparent antenna in the lens which would allow having windings over the pupil). In our system, the eyewear with the primary antenna is assumed to be firmly strapped to the head to ensure that the device does not move during use (so the only variable parameter is the orientation when the eye rotates). When the primary and secondary coils are not parallel, this efficiency is further reduced. The rotation of the secondary coil (w. r. t. the primary antenna) from 0° to 16° leads to a 30% loss but the optical power stays always above the laser threshold, and does not impact the VCSEL spots detection system.

### 3.2. Impact on beam direction

**Fig 2:**
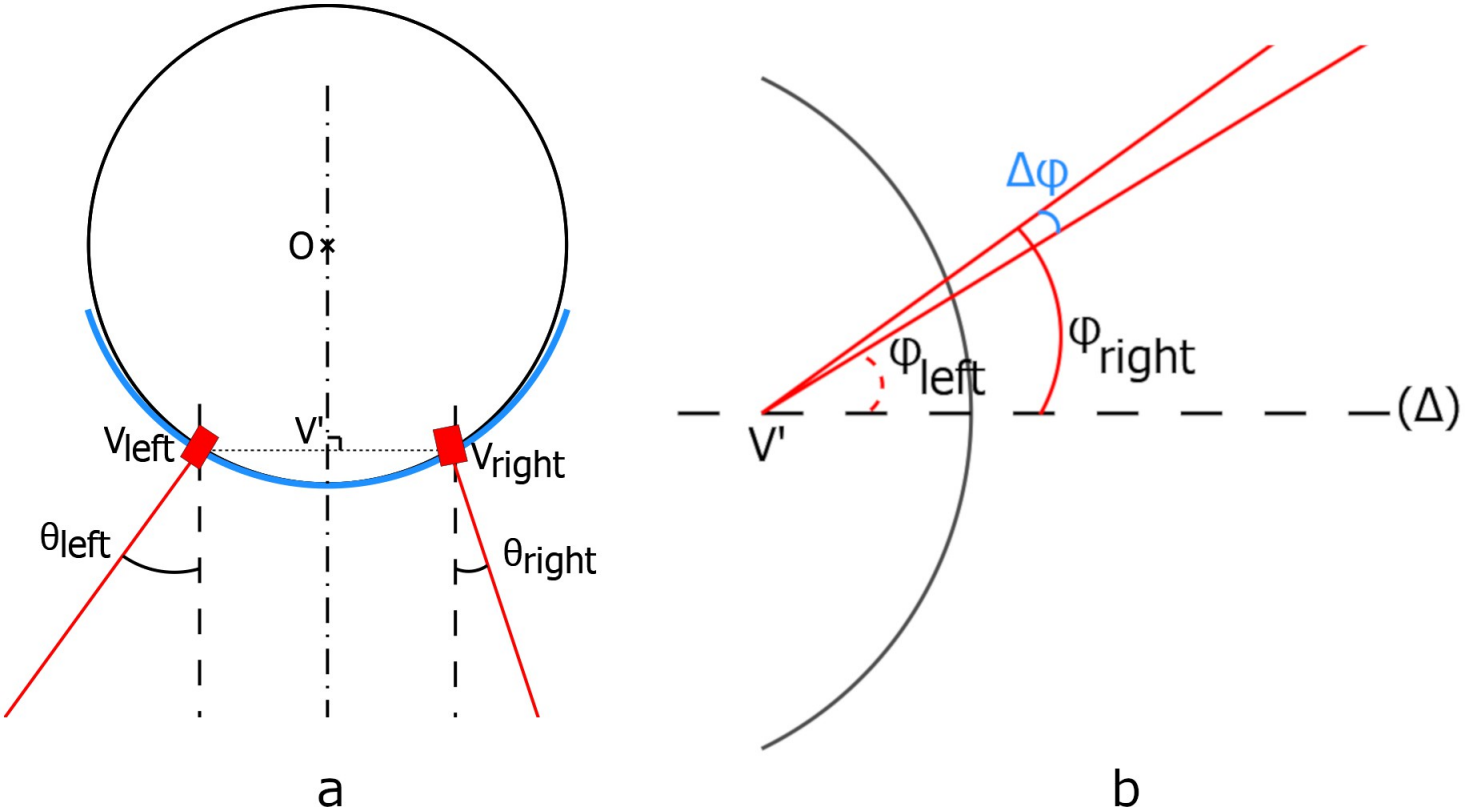
(a) CLP top view with the horizontal angular separation; (b) Side view with the vertical angular separation.

The encapsulation process creates strains on the circuit which is bent due to the curved shape of the contact lens. As a result, the beam direction with respect to the normal of the pupil may vary from one VSCEL to the other. We denote respectively θ and φ the angles between the beam and the normal to the pupil in the horizontal and vertical planes (Fig 2). The angular separation between the two VCSELs in the horizontal plane is thus Δθ = |θ_left_−θ_right_| where left and right denotes the left and right VCSEL. Similarly, the angular separation between the two lasers in the vertical plane is Δφ = |φ_left_−φ_right_|.

**Fig 3:**
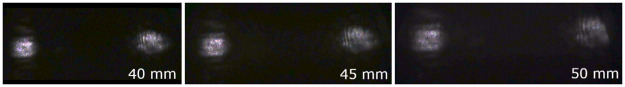
Pictures of the spots on the paper screen recorded at three distances of the CLP: 40 mm, 45 mm, and 50 mm.

These angles were measured using the following procedure. The CLP is positioned on the eye so that both VCSELs are horizontally aligned. For a chosen VCSEL, the spot emitted is projected on a paper screen. On the backside of the screen, a camera records a picture of the spot. The exposure time of the camera is initially set to avoid pixels saturation. Measures are made for different directions of gaze around the estimated beam propagation direction, and for three CLP-screen distances. Rotations around this direction are achieved degree by degree using a rotative plate Newport URS100-PP, with an absolute precision of 0.03°. For each angle, three pictures of the VCSEL spot are recorded (one for each three CLP-screen distances, cf. Fig 3). Spot’s centroid coordinates are computed, as well as the x and y dispersion for the spot position over the three distances. A minimum dispersion of the spot centroid coordinates indicates that the beam propagation axis is found. Then, left and right rotations of 0.5° are achieved around this angle to confirm this value.

**Fig 4:**
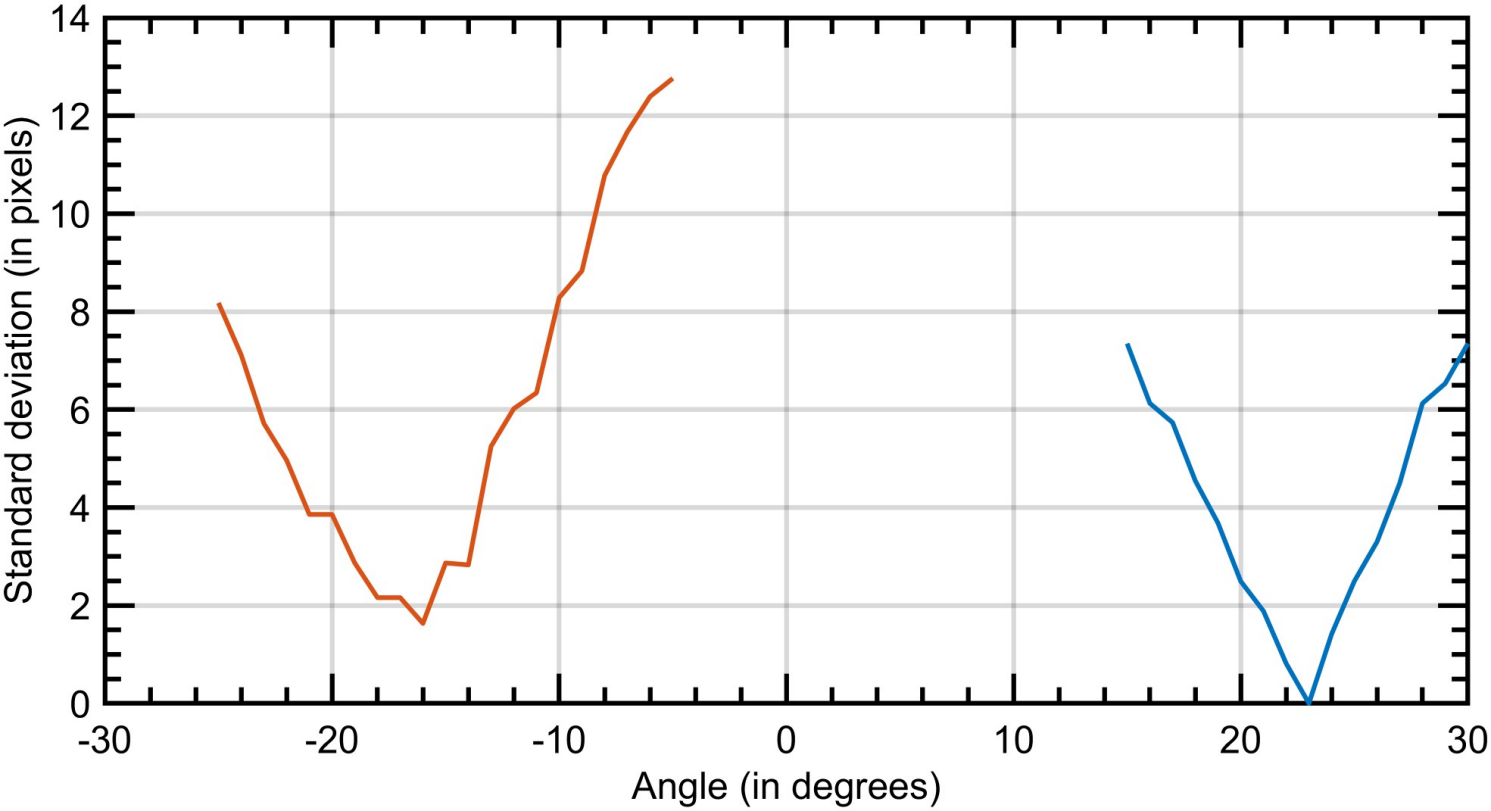
Abscissa: Beam direction (horizontal). Ordinate axis: standard deviations (in pixels) of the centroid positions in x over three CLP-screen distances, for a CLP sample. When this value is minimal, the screen is considered normal to the direction of emission of the VCSEL.

The same procedure is repeated with the second VCSEL. Fig 4 illustrates the process for the calculation of θ. Measurements are synthetized in Table 1. The horizontal angular separation is between 39° and 46° with an average of 42° depending on the sample. These variations are due to our manual encapsulation process. Then the CLP is rotated by 90° on the mock-up eye, so that the VCSELs are vertically aligned, and the operation is repeated to measure the vertical angular separation. A well-chosen angular separation for the two VCSELs could be leveraged to design a complete spot’s detection device implementation with PSDs. The encapsulation process should be improved to set the desired angle (see Section 5).

**Table 1:** Beam direction (according to Fig 2) for the non-encapsulated circuit and the three CLP samples.

	Horizontal θ		Vertical φ	
	Left VCSEL	Right VCSEL	Left VCSEL	Right VCSEL
**bare circuit**	3°	1°	1°	3°
**CLP I**	24°	-16°	7°	12°
**CLP II**	23°	-16°	-1°	3°
**CLP III**	35°	-11°	1°	8°

### 3.3. Impact on the VCSELs divergence

**Fig 5:**
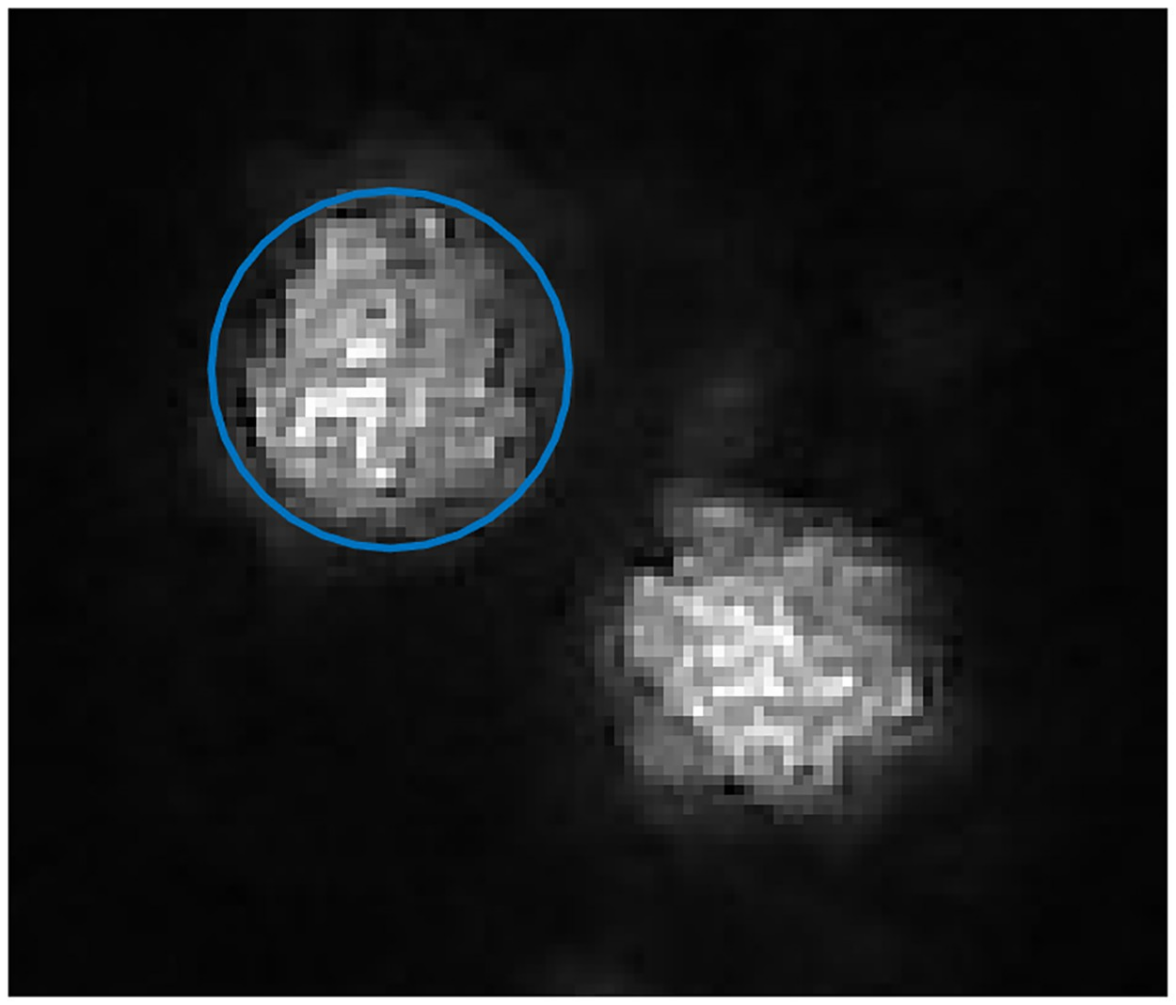
Example of waist detection (blue circle).

Once determined the beam propagation axis for each VCSEL with respect to the normal to the pupil, the following method was used to assess the impact of encapsulation on the VCSELs divergence. One VCSEL is positioned so that its beam is normal to the camera’s plane. A paper sheet is placed between the camera and the VCSEL and a picture of the spot on this sheet is taken for three sheet positions, as in section 3.2. The exposure time of the camera is again set to avoid pixels saturation. The beam is assumed to be Gaussian. The images are then processed to calculate the diameter of the spot defined as the diameter of the circle centered on the spot weighted centroid and containing 86.5% of the emitted power. An example of waist extraction is presented in Fig 5. For each distance, a waist is computed and the corresponding divergence angle deduced. The retained angle value is defined as the mean of these three angles. We notice that the encapsulation causes a slight increase in the divergence of each VCSEL. The half-angle of divergence is 4° for non-encapsulated VCSELs (in agreement with the manufacturer datasheet) and 5.5 ± 0.5° when encapsulated.

In conclusion, the encapsulation has an impact on the beam characteristics (power, direction, divergence) but this one is relatively mild and quite similar over the three encapsulated samples despite the manual manufacturing process.

## 4. Detection of the VCSELs’ spots

Two sensor options have been studied to detect the VCSELs spots: a digital camera and a PSD.

### 4.1. Camera version of the eye-tracking system

**Fig 6:**
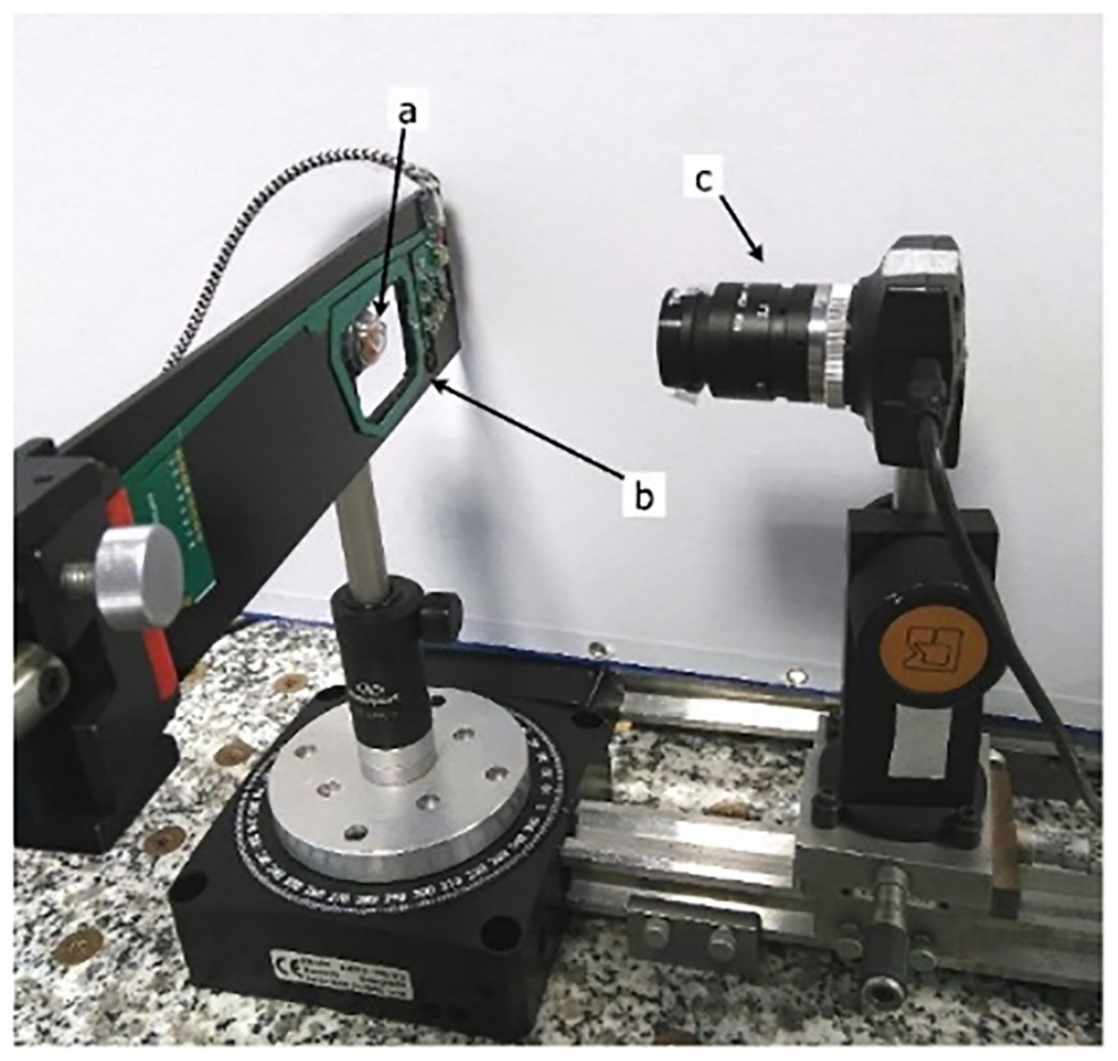
Camera characterization bench. a) CLP on the mock-up eye, b) primary antenna. c) CMOS camera.

The first way to detect a spot is using a camera. Unlike the characterization stage, the camera is now focused on the emission surface of the VCSEL. A band pass filter (Thorlabs FB850-10 centered on the VCSEL’s emission wavelength with a 10nm FWHM) is used to filter out visible light and facilitate spot detection). The CLP is mounted on a plastic mock-up eye, fixed on the same rotative plate Newport URS100-PP as in section 3.2. The camera (IDS UI-1245LE-M-GL) has a CMOS sensor with a resolution of 1280×1024 pixels (pixel size is 1.3 × 1.3 μm^2^) and is used with a 25mm objective. It operates at (f/16) to avoid the blur caused by the displacement in depth of the VCSELs dues to the CLP’s rotation. The camera is placed at a distance of 12.5 cm from the CLP (Fig 6).

As presented in our first study [11], the eye tracking system resolution is directly related to the number of pixels scanned by the spot image on the camera during an eye rotation. Since the camera images on a 2D plane the VCSEL displacement which occurs in 3D, this displacement is smaller at large eccentricities (i.e. eccentric direction of gaze), thus impacting the tracking resolution. Two series of measurements (in the horizontal plane) have been recorded for two ranges of eye rotation: the first between -3° and 3°, the second between 13° and 16°, by step of 0.2° (further away than 16°, the subject would rather turn the head than the eye [12]). For both angular domains, the relation between spot displacement and direction of gaze was strongly linear (r^2^>0.95) which means that once calibrated, the direction of gaze could be directly inferred from the spot position on the sensor. The slope coefficient for respectively the left VCSEL and the right VCSEL was 14.3 and 13.7 pixels / degree in the central domain, and 12.2 and 14.2 pixels / degree in the eccentric domain. The model is thereafter used on test pictures taken at different angles. The accuracy can be calculated as the maximum error between the known real rotation angle and the angle computed by the model applied to test data. The measured achievable accuracy is of 0.11°. It is worth noting that only a small part of the sensor was used. This means that provided another optics, a better resolution could be achieved or alternatively, a cheaper smaller sensor could be used.

### 4.2. PSD version of the eye-tracking system

**Fig 7:**
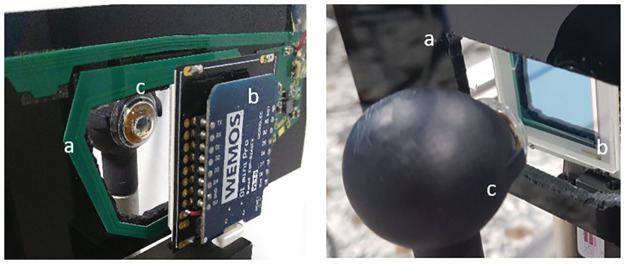
PSD eye-tracking test bench. a) primary antenna. b) PSD, c) CLP mounted on the mock-up eye.

**Fig 8:**
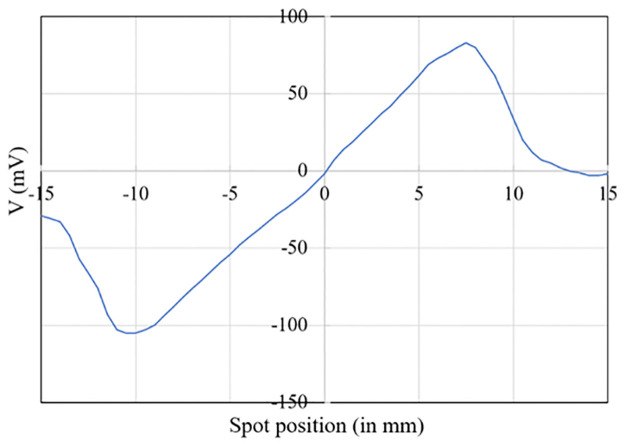
Voltage measurement as a function of the spot position on the PSD (after offset correction).

The second way to detect a spot is with a PSD. The VCSEL is then considered as a pointer. The PSD has no focusing power and the beam divergence must be considered. PSD refers to a component based on silicon PIN diode technology, used to measure the position of the integral focus of an incoming light. It uses the effect of the lateral division of the generated photocurrent. A light spot on the PSD is converted into a continuous electrical signal related to the focal position of this spot. The position of the spot on the PSD is derived from the relationships between four output currents (two for x and two y), expressed as follows [13]:

$$\begin{array}{l} x=\frac{L}{2}\frac{I_{x2}-I_{x1}}{I_{x2}+I_{x1}}\\ y=\frac{L}{2}\frac{I_{y2}-I_{y1}}{I_{y2}+I_{y1}} \end{array}$$ (1)

where I_x1_, I_x2_, I_y1_ and I_y2_ are the electrode photocurrents, L is the length of the PSD active area and x and y stand for the laser spot positions. We first used a PSD OSI Optoelectronics DL-20-C, consisting in a 2D squared silicon plate, 20 mm×20 mm large. The test bench is depicted Fig 7. The PSD is linear over 68% of the central surface, with a precision of 200 μm (Fig 8).

Fig 8 shows the PSD response after offset correction.

The resolution depends on the linearity of the interelectrode resistance. The PSD resolution is improved by increasing this resistance with a reduction of both amplifier and shot noise [13]. It relies on the assumption that the two analogue photocurrents for one direction measured at the pins of the PSD are amplified with two identical amplifiers, which is difficult to be achieved at a low cost. Furthermore, the resolution increases with the CLP-PSD distance provided that the beam divergence is limited, which is not the case in the current CLP configuration. We tested two PSD positions: at 1.5 and 2 cm from the CLP. During this experiment only one was VCSEL was used, the other one was blocked. At these distances, no saturation occurs. The spot size is smaller than the sensor size and the PSD can convert directly the incident light spot into continuous position data. In the camera configuration, an objective lens is required otherwise the spot on the CMOS sensor would be too large and the direction of gaze impossible to calculate.

**Fig 9:**
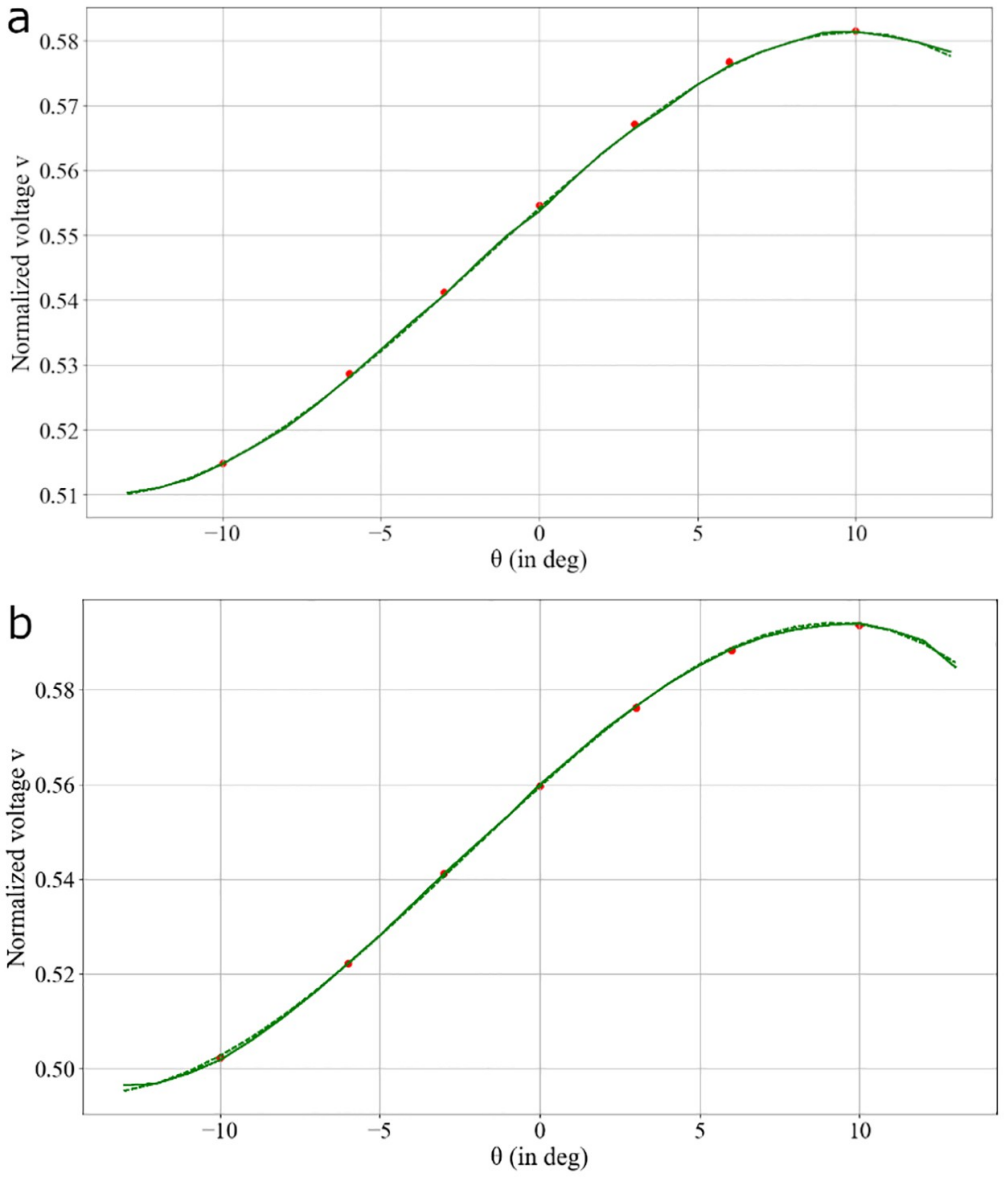
PSD measurements as a function of gaze direction for two CLP-PSD distances: (a) 1.5 cm and (b) 2 cm. The continuous line represents the experimental data and the dashed line the fitted model.

The current at one electrode is copied by a current mirror. A measurement resistance is placed between the pins and ground to convert it into a voltage, so that voltages can be measured. The voltage corresponding to the sum of photocurrents is used to normalize the electrode voltage, to prevent intensity variations, and improve the PSD linearity when the spots reach the boundaries. The ADC used to acquire the voltages from the PSD circuit allows to save 6000 measurement points per second. In order to reduce the noise due to our custom electronics, and because high-end commercial head-mounted eye-trackers rarely work faster than 120 Hz, the data was averaged over a 83ms window (so 50 voltage values). Experimental results with respect to the angle of gaze are presented in Fig 9.

The PSD response appears linear between -10° and 6° and becomes nonlinear when approaching the device boundaries. To directly associate a direction of gaze (θ) to the PSD voltage, a polynomial regression of degree 3 was computed over the range [-10°,10°]. The best achievable accuracy for the two distances was 0.33°. In addition, the standard deviation on measurement error was 0.005° which means that the width of the window used was large enough.

To evaluate the impact of our electronics on the eye-tracker’s performances, the measures have been compared with the one’s obtained with a PSD from Hamamatsu Photonics together with its dedicated electronics. The PSD tested is the S1880, with a linear surface of 12 by 12 mm^2^, and the processing circuit is the C4674-01. The positions in X and Y are respectively directly measured by one output of the processing circuit. The voltage corresponding to the X-position has been also measured with this PSD but has not been normalized here. The central linear part of the PSD is corresponding to angles between -12° and 4°. The processing circuit allows to measure less noisy values of voltages. The averaging is then not necessary anymore in that case and an accuracy of 0.25° can be reached without averaging the data. This means that the PSD (S1880) can theoretically work at a frequency of 8000 Hz, which is far beyond the specs of current high-end eye-trackers.

## 5. CLP with a predetermined designed VCSEL orientation

**Fig 10:**
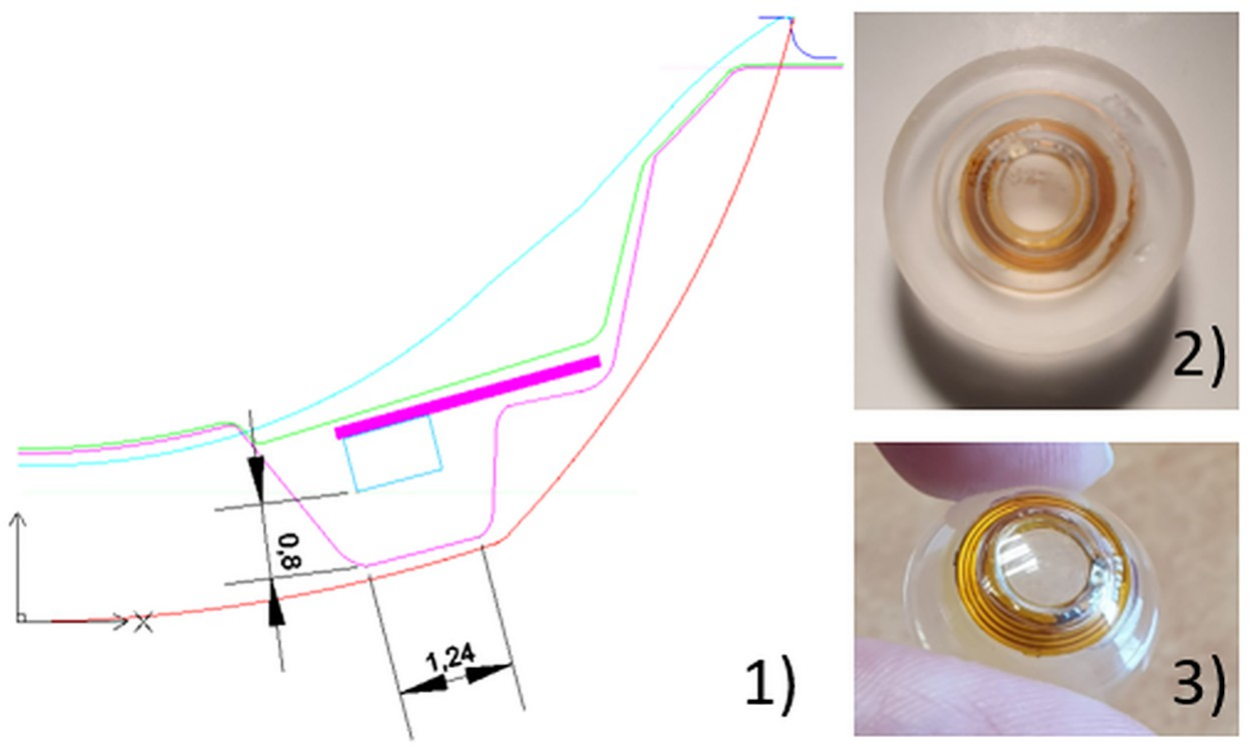
New manufacturing design to control VCSEL eccentricities. Top right image: the CLP in the lens blank before lathing. Bottom right: the final CLP. Left: cross-section of the CLP where a) is the front surface and b) the VCSEL and circuit housing.

From the previous experiments, we have seen that the beam divergence is detrimental to the PSD option (If the PSD is moved at 60 mm from the VCSEL, the surface illuminated by the spot covers almost 50% of the PSD’s surface and the detection error increases to 0.8°.), imposing a proximity of the PSD to keep a small spot but with penalties on angular resolution (the difference between 1.5 and 2 cm was not noticeable here). This can be solved by controlling the beam divergence, etching for instance an optical power inside the CLP in front of the VCSEL. Similarly, whether for the camera and PSD implementations, beam eccentricities should be chosen and controlled to optimize CLP embodiments in glasses or AR headsets and improve the system performance (for instance to allow placing the sensors as close to the eyes as possible without reducing the FOV). This can also be achieved during the manufacturing process by etching an empty cavity slot in front of the VCSEL with a given orientation (Fig 10(1)).

The contact lenses used to encapsulate the VCSEL and associated electronics are scleral ones with a diameter of 16.50 mm. In [7], the electronics was preliminary sandwiched between two half-lenses via a bonding process. Each half-lens was lathed separately. The bottom one would receive the circuit and the upper one would close it. As a result, a cavity, filled with air or glue, would be present on the optical axis, possibly impacting vision. In order to better control the positioning of the VCSELs and improve the optical quality a new manufacturing process was set-up. The electronics is encapsulated between 2 pre-manufactured pallets (see Fig 10(2)) which are sealed together before the upper and lower surfaces are polished to manufacture the lens curvatures to obtain the final wearable contact lens as presented (Fig 10(3)).

**Fig 11:**
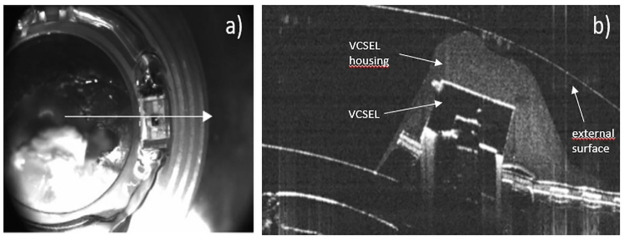
New manufacturing design to control the VCSEL eccentricity. a) top view CLP of the CLP, the arrow illustrates the meridian of the cross section depicted in b); b) cross-section showing the VCSE in its housing.

To test that we could control the VCSEL orientation by etching an empty cavity in front it, a CLP was manufactured according to the design illustrated (Fig 10(1)) with a chosen VCSEL orientation of 25°. Fig 11 presents the resulting element. Fig 11b shows a cross section of the device recorded with optical coherence tomography (Optotvue ivue-80) along the meridian illustrated in Fig 11a. As can be seen, the quality of the lathing process allows to create a controlled cavity above the VCSEL that could also contain an additional optical element. The direction of emission of the VCSEL was measured at 22° (with the same process as in section 3.2) which is in agreement with the theoretical value (25°).

## 6. System implementations

The two spot detection configurations depicted in the previous section have been implemented into an eyewear and are presented hereafter.

### 6.1 System with a camera

**Fig 12:**
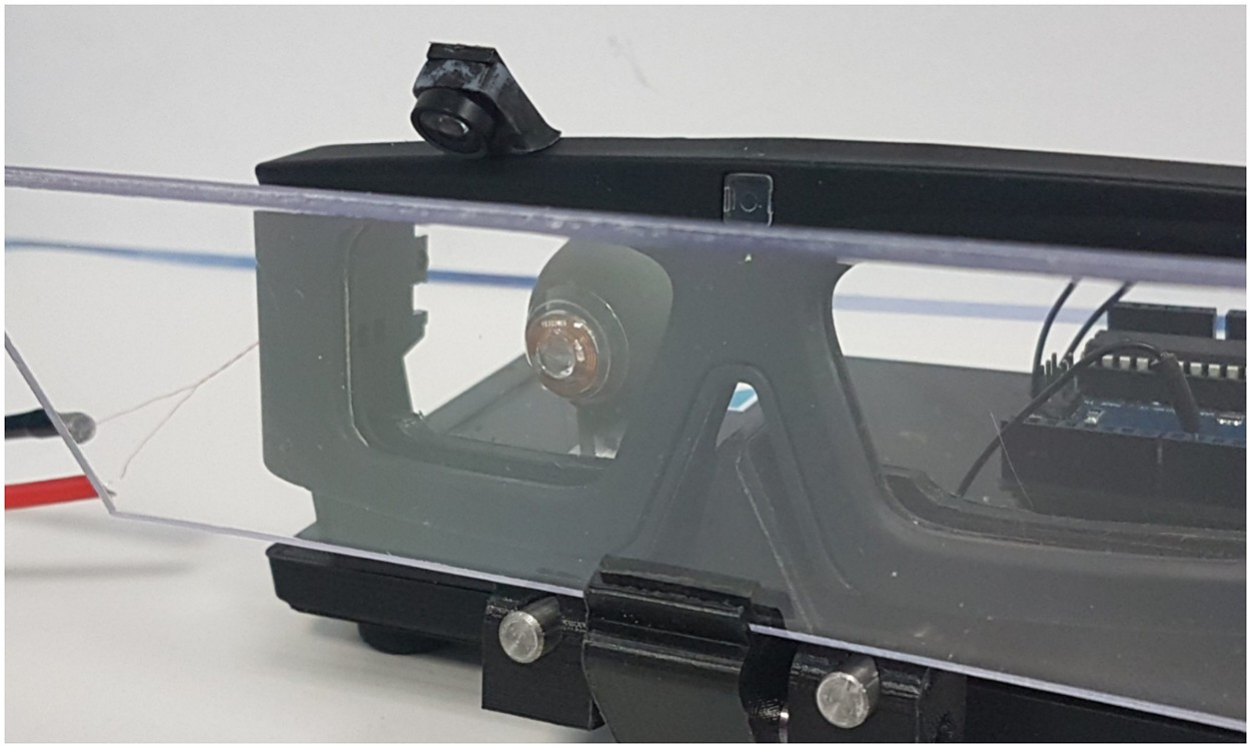
Eyewear in the camera configuration. The camera is mounted on top the eyewear frame. The beam splitter allows positioning the camera virtually in front of the eye.

In the final system, the camera is closer to the CLP than on the testbench to be easily embedded in the host IVAS. The choice of the camera is conditioned to several parameters: the focal length and the f-number to be able to image the VCSELs at close distance, and the resolution of the CMOS sensor as a limiting factor for the system precision. In Fig 12 we used a camera from Omnivision (OV2640) with a resolution of 1600x1200, FOV = 60°, 2.2 × 2.2 μm pixels and similar f-number as in section 4.1.

In Fig 12, the camera is placed above the eye and a beam splitter is used to align the camera with the line of sight when the subject looks at a target in front of him at far range. A mechanical support allows adjusting the position and tilt of the beam splitter. The beam splitter coating allows maximizing reflections at 850 nm and minimizing the ones in the visible range Alternatively, a single camera could be used for both eyes, thus avoiding any synchronization issues between the two cameras but at the expense of a more complex optics [14].

### 6.2 System with a PSD

**Fig 13:**
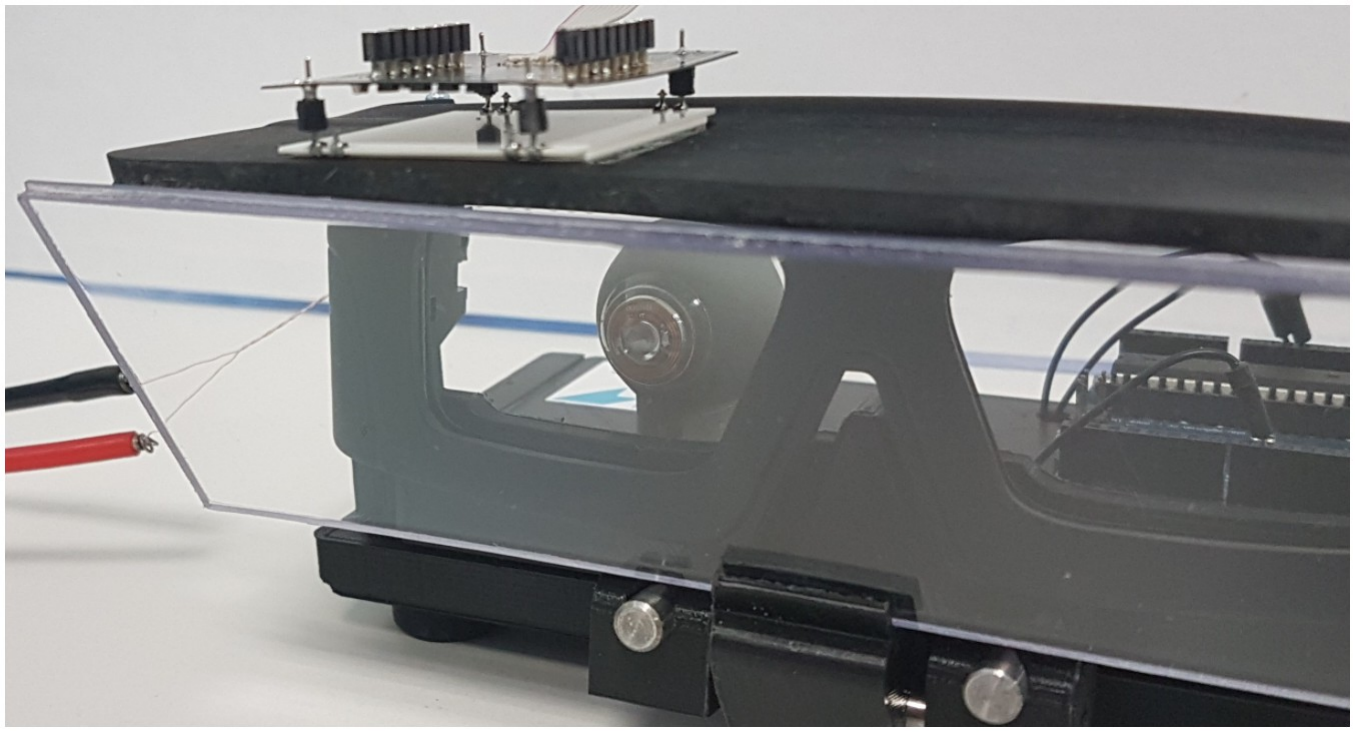
PSD compact eyewear. PSD is mounted on top of the eyewear.

**Fig 14:**
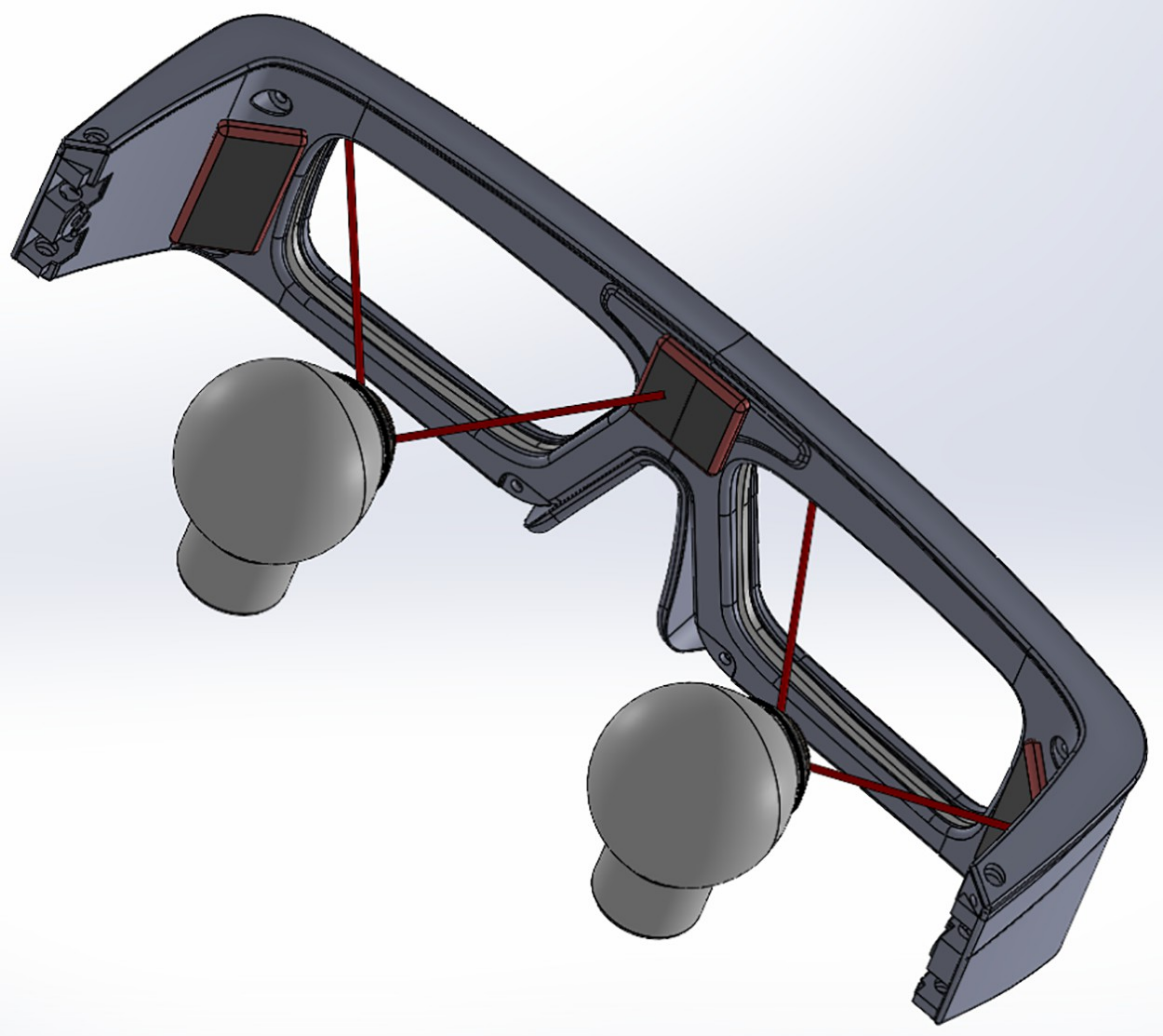
The angular orientation of the VCSEL on the contact lens can be chosen to facilitate positioning of the PSDs close to the eye and to extend the gaze range over which the eye is tracked.

In this configuration, the PSD is positioned close to the eye to avoid the loss of precision due to the spot spreading caused by the VCSEL divergence. Commercially available PSD are not transparent so cannot be placed directly in front of the eye (however a new generation of transparent graphene-based PSD [15] provides promising ultra-long carrier diffusion length which facilitates a large active area). Therefore, using conventional PSD results in various embodiments. A first solution consists in using a beam-splitter option as in 6.1 to place the PSD above the eyewear (Fig 13). However, the distance is large according to the beam divergence and the PSD should be placed closer to the CLP. This means that the use of two PSD instead of one, located on each side of the direction of view would be preferable as described in Fig 14. This configuration would benefit from the double VCSEL solution and their angular separation, so that no matter the gaze direction, there would be at least one of the two PSDs receiving one (and only one) of the laser beams. This strategy enables us to place the opaque PSD detectors as close as possible to the eye without reducing significantly the field of view. The angle between the spots being known, the gaze direction can then be easily calculated. (Methods to detect multiple spots position on a PSD have been proposed but this does not happen in the configuration we described).

The distance between both PSDs would have to be chosen to let a sufficient field of view while limiting the resolution loss due to the beam divergence. FOV with spectacles is large (> 90°), making this solution more appropriate for head mounted displays such as AR glasses where the FOV is closer to 30° horizontally (17.5° vertically). This consideration emphasizes the importance of exploiting the VCSEL beam eccentricities. An example of use of controlled emission angles on the lenses is presented Fig 14. The angles on the CLPs are chosen so that there is at least always one VCSEL spot that is detected. If the wearer looks in front of him, the right VCSEL on the left eye will impact the central PSD; if the wearer looks left, the left VCSEL on the left eye will hit the lateral PSD, etc.

Finally, little post-processing is required with the PSD to extract the spot position, thus allowing to track the gaze direction at very high rates (the PSD used in this study provides 8000 measurements per second).

## 7. Conclusion

We have demonstrated how a VCSEL pair CLP can be used efficiently to analyze the gaze direction. We have investigated two complementary detection schemes: one using a digital camera, another a PSD. We have set up a common characterization protocol to provide reliable measurements of the angular values. We have demonstrated that an angular accuracy of 0.11° with a camera and of 0.25° with a PSD could be reached, which would easily outperform the performances of existing head-mounted eye-trackers [16]. These performances were obtained on a highly controlled test bench and performances in real conditions will be undoubtedly poorer than on the artificial eye and assessing it will be our next research objective now that we have demonstrated a proof of concept.

In terms of compactness, the use of a VCSEL pair whose beams direction can be predetermined during the manufacturing process enables to place the sensors (camera or PSD) very close to the eye without reducing the field of view, allowing for instance to remove the beam splitter of Figs 12 and 13 and making the system easily integrable in existing eye-wears and AR headsets. It could also be used to track the eye over a wider range of gaze.

In terms of speed, the PSD outputs two photocurrents to detect the incident beam position while the camera requires some image processing. Hence, the PSD allows to track the gaze direction much more rapidly than with conventional video-based eye trackers. In contrast camera-based systems can take advantage of a large range of existing image processing techniques to compute laser beam positions (e.g. from centroid, like here, to deep learning [13, 17]).

In terms of angular resolution, we obtained almost similar accuracy values for the gaze direction, with the 2 systems. However, we did not fully take advantage of the CMOS sensor size so it may be easier to achieve higher accuracy with the camera system and the final choice between PSD and camera is mainly conditioned by the host IVAS embodiment constraints.

In conclusion, in both cases we demonstrated that engineering appropriately the beam direction of a pair of VCSELs pointers embedded into an autonomous contact lens provides a new range of highly integrated and accurate eye-tracking systems, being easily incorporable in many IVAS devices.
